# Contrast-enhanced mammography in the setting of neoadjuvant therapy from a multidisciplinary perspective

**DOI:** 10.1186/s13244-026-02294-5

**Published:** 2026-07-15

**Authors:** Lígia Pires-Gonçalves, Conceição Leal, António Guimarães-Santos, Ana Teresa Aguiar, Miguel Abreu, Donzília Brito, Rui Manuel Henrique

**Affiliations:** 1 Department of Radiology and Cancer Biology and Epigenetics Group – Research Centre (CI-IPOP), Instituto Português de Oncologia do Porto (IPO-Porto), 4200-072 Porto, Portugal; 2 Department of Pathology, Instituto Português de Oncologia do Porto (IPO-Porto), Porto, Portugal; 3 Department of Radiology, Instituto Português de Oncologia do Porto (IPO-Porto), Porto, Portugal; 4 Department of Medical Oncology, Instituto Português de Oncologia do Porto (IPO-Porto), Porto, Portugal; 5 Department of Surgical Oncology, Instituto Português de Oncologia do Porto (IPO-Porto), Porto, Portugal; 6 Department of Pathology and Cancer Biology and Epigenetics Group—Research Centre (CI-IPOP), Instituto Português de Oncologia do Porto (IPO-Porto), Porto, Portugal; 7 Department of Pathology and Molecular Immunology, Institute of Biomedical Sciences Abel Salazar (ICBAS), University of Porto, Porto, Portugal

**Keywords:** Breast cancer, Contrast-enhanced mammography, Neoadjuvant therapy, Precision medicine, Pathology

## Abstract

**Abstract:**

Contrast-enhanced mammography (CEM) is a morphofunctional imaging technique that is gaining interest and acceptance in clinical practice. With increasing use of neoadjuvant therapy (NAT) in breast cancer and of precision medicine, the need for equitable access to appropriate high-quality advanced imaging for monitoring response has become ever more relevant. CEM is an imaging alternative for NAT monitoring, with encouraging initial results and a good logistical profile. In this educational review from a practical perspective, we explore key aspects for response assessment of NAT and CEM roles in this setting, based on relevant literature and the authors’ clinical experience. Pearls and pitfalls, as well as opportunities and challenges for NAT monitoring with CEM, will be approached from the perspectives of the radiologist, the oncologist, the surgeon, the pathologist, and the patient.

**Critical relevance statement:**

The early experience with CEM in the NAT setting is positive, encouraging its further development, not only from the radiologist's view but also from a broad multidisciplinary perspective.

**Key Points:**

- CEM seems to be non-inferior to MRI for response evaluation and preoperative re-staging after NAT.
- CEM may predict and monitor in vivo chemosensitivity during NAT.
- CEM may allow a greater patient flow rate compared to MRI.

**Graphical Abstract:**

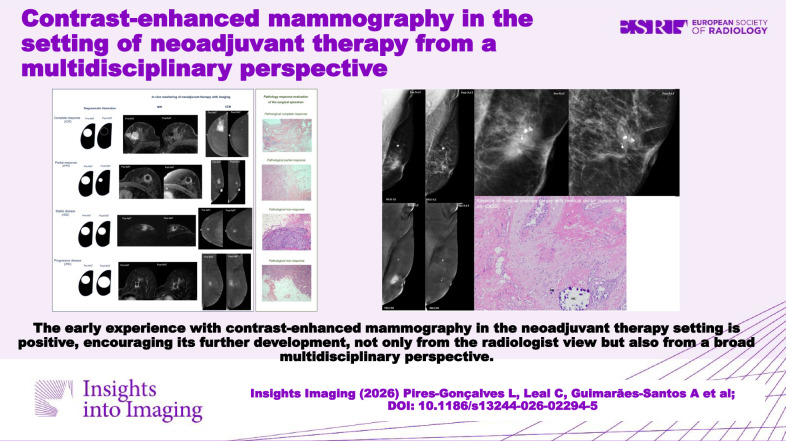

## Background

Neoadjuvant therapy (NAT) is increasingly used for breast cancer treatment [1, 2]. Compared to adjuvant chemotherapy, NAT offers comparable long-term distant and local-regional cancer control [3, 4], facilitates breast and axilla-conserving surgery, and can convert patients with inoperable tumors into surgical candidates [1, 4, 5]. In patients with operable disease, NAT is commonly used for human epidermal growth factor receptor 2 (HER2) positive and triple-negative breast cancers larger than 2 cm (≥ cT2) and/or with positive lymph nodes (≥ cN1), and may also be considered for smaller, node-negative, HER2-positive or triple-negative breast cancer [1–5]. Furthermore, NAT can be considered in patients desiring breast conservation with tumors large relative to breast size or in whom surgery is delayed, regardless of the molecular subtype. NAT can also aim to render operable patients with inoperable tumors, such as inflammatory breast cancer or bulky or matted axillary nodes (cN2, cN3) [1–5]. Additionally, the response to NAT provides important prognostic information in an individual patient, guiding adjuvant systemic treatment, as well as the unique opportunity of in vivo tumor response monitoring, enabling real-time insights into tumor evolution and response.

Presently, imaging is used to exclude disease progression during NAT and to evaluate residual disease after NAT, allowing effective surgical planning. Comparisons of clinical breast examination, mammography, ultrasound, and MRI, have found MRI to be the most accurate method for determining tumor response and residual tumor [6, 7]. The superior performance of MRI is mainly related to its ability to depict functional data, such as perfusion.

Contrast-enhanced mammography (CEM) is an emerging technique that can evaluate both morphological and functional data, similarly to MRI. The early experience with CEM for NAT response assessment is encouraging [8–16]. CEM entails several logistical advantages. Compared to MRI, CEM is faster, less expensive, more patient-friendly, potentially more available, and has fewer contraindications [17, 18].

With the increasing use of NAT in breast cancer patients, the need for equitable access to appropriate high-quality advanced imaging for evaluating tumor response gains momentum. Presently, CEM is an alternative for NAT assessment, particularly valuable when MRI is unavailable, has limited access, or is contraindicated. CEM's broader use might contribute to a more equitable access to high-quality advanced imaging.

In this educational review from a practical perspective, we explore key features for response evaluation of NAT and CEM roles in this setting, based on relevant literature and the authors’ clinical experience. We will address CEM technical issues, review critical aspects of NAT and of response assessment with CEM, its correlation with MRI and pathology, as well as provide a reflection on CEM opportunities and challenges from the radiologist, the oncologist, the surgeon, the pathologist, and the patient perspectives. We aim to contribute to a more confident use of CEM in the NAT clinical setting.

## CEM technical review

**Fig. 1:**
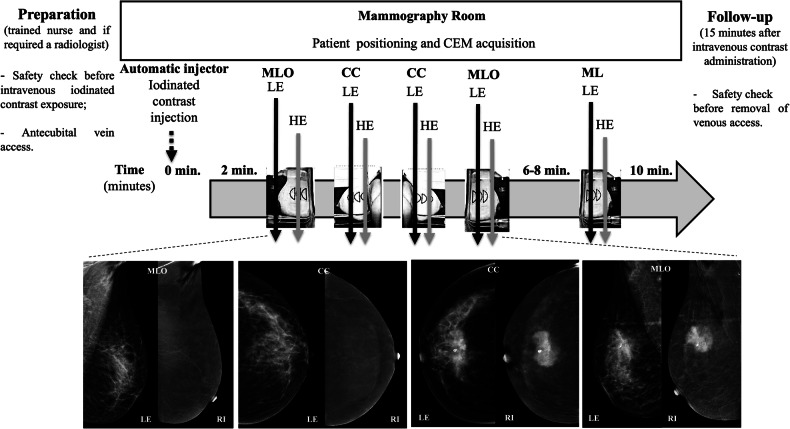
Diagram of the patient workflow for CEM acquisition. The horizontal arrow represents the time window of 10 min in which a full CEM (at least four views) is ideally acquired. The iodine-based contrast agent is administered at time point zero (dashed arrow). We use an automatic injector to administer Omnipaque 350^®^, a nonionic low-osmolar iodinated contrast at a concentration of 350 mg iodine/mL, (iohexol, GE Healthcare), at a dose of 1.5 mL/kg and a rate of 3 mL/s followed by 20 mL of saline flush at the same flow rate. A delay of at least 2 min between contrast injection and CEM acquisition is required. Each CEM acquisition requires a pair exposure to low-energy and high-energy, both obtained in the order of seconds and within one compression (vertical black and gray arrows). Six to eight minutes after intravenous contrast, a delayed view can be acquired for the pathologic breast. After image automatic processing, a low-energy (high resolution/morphologic) image and a recombined (post-processed iodinated map/functional) image are retrieved in each view for clinical assessment. MLO, mediolateral oblique view; CC, craniocaudal view; ML, mediolateral view; LE, low energy; HE, high energy; RI, recombined image/functional image; min., minutes

First commercially introduced in 2011, CEM is currently widely used for the detection and evaluation of breast lesions in several clinical settings and has more recently been used to guide breast biopsies [19]. CEM uses multiple views, after a single injection of radiopaque iodinated contrast agent, to study the morphology and the perfusion of the breast (Fig. 1). At our institution, prior to CEM, a nurse, and if required a radiologist, explain the exam to the patient and perform a safety check (Fig. 1). Pregnancy, untreated hyperthyroidism, previous history of iodinated contrast agent adverse reaction and of kidney disfunction, as well as creatinine blood values, if available, are specifically checked. Alternative imaging with MRI is considered in patients with breast implants and with a previous history of an iodinated contrast agent adverse reaction. Patients at risk of contrast-associated acute kidney injury require renal function assessment before CEM. Contrast-associated acute kidney injury is defined as a decrease in renal function within 48 h after exposure to an intravenous iodinated contrast agent [20]. It is uncommon and has progressive risk with increasing stages of chronic kidney disease [20].

The ideal temporal frame for CEM acquisition is 2–10 min after intravenous contrast, but iodinated contrast breast uptake has been demonstrated from 2 min to 20 min after contrast administration [21–24]. Like conventional mammography, CEM is a planar technique that requires two views to infer the lesions’ spatial location. CEM exposure time depends on breast size and settings used, generally varying between 4 s and 10 s/view [21–24]. Despite a previous report that the order of the acquisition of the views is indifferent to image quality [23], large sample and prospective studies comparing the impact of different acquisition orders on diagnostic performance would provide valuable evidence for protocol optimization [19]. In our feasibility assessment for CEM implementation in the NAT setting, the average time required for acquisition of a mediolateral oblique and a craniocaudal view in bilateral CEM was 5 min (range: 4–7 min) and in unilateral CEM was 3 min (range: 3–5 min), measured from contrast injection to final mammographic view [25]. This information is important for adequate scheduling.

**Fig. 2:**
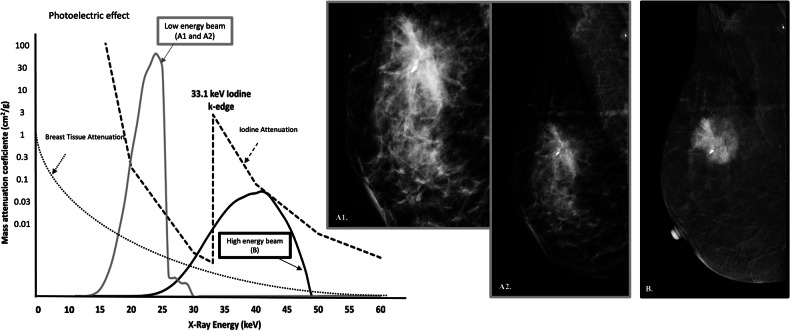
Brief description of the physics inherent to CEM. The detection of iodinated contrast uptake with CEM is possible due to a photoelectric effect that depends on the X-ray absorption differences between iodine and breast tissue. The energy of the X-ray beams, as well as the absorption k-edge of iodine (33.1 keV), are in the average range of the X-ray beam of mammography. First, the low-energy image is acquired using tube voltages varying between 26 and 30 kVp. Therefore, although iodinated contrast is already within the breast at this point, its mean energy falls below the k-edge of iodine (33.1 keV), and the image obtained is equivalent to a normal mammogram (**A1**, **A2**). The high-energy images are acquired second, using tube voltages of 44–49 kVp and extra X-ray filtration to ensure that the X-ray beam spectrum is almost entirely above the k-edge of iodine. Although the high-energy image contains relevant information, this cannot be perceived by the human eye. A weighted subtraction of the low- and the high-energy images produces a recombined image (iodine map) that maximizes the conspicuity of iodinated contrast distribution in the breast, while minimizing the structured noise of non-enhancing fibroglandular tissue (**B**). The image A1 corresponds to the amplification of **A2**. **A1** perfectly exemplifies the high spatial resolution of the low-energy images (ten times the spatial resolution of breast MRI) that allows adequate assessment of microcalcifications

**Fig. 3:**
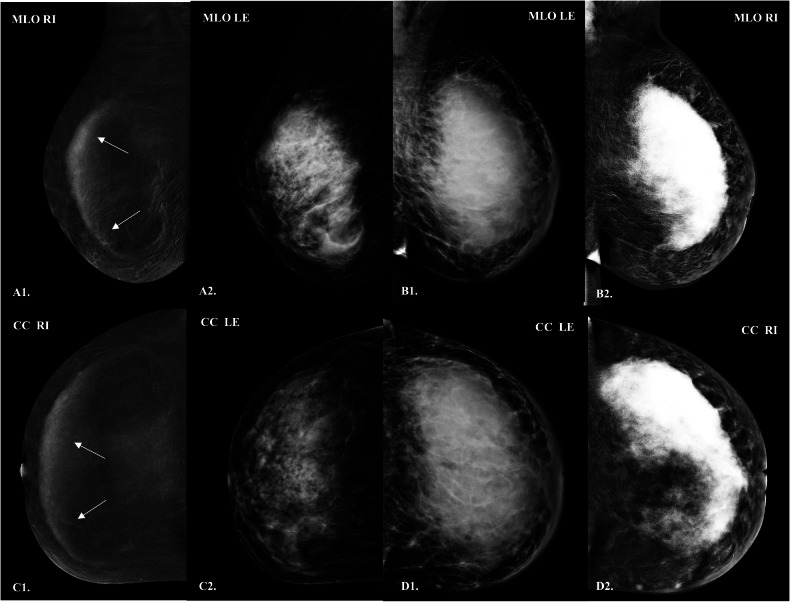
CEM in a 33-year-old woman before NAT (baseline study). **A1**–**C2** The normal morphology (**A2**, **C2**) and background parenchymal enhancement/normal perfusion (**A1**, **C1**) of the breast are shown in the non-pathological side (right breast). In this example, the breast was extremely dense and exhibited mild background parenchymal enhancement. **B1**–**D2** The recombined images clearly demonstrate the tumoral extent in the pathological breast (left breast) (**B2**, **D2**). The cancer infiltrated the totality of the left breast parenchyma with breast edema and diffuse skin thickening. Notice the superiority of recombined images compared to conventional mammography for demonstrating tumoral extent, even in the setting of extremely dense breasts, as depicted in this example. Also, notice the breast-within-a-breast artifact (arrows) that can hamper CEM recombined images reading. MLO, mediolateral oblique view; CC, craniocaudal view; LE, low energy; RI, recombined image

In Fig. 2, the physics behind CEM is summarized. The high spatial resolution of the digital detector (low-energy images) reveals lesion details with approximately ten times the spatial resolution of MRI, similar to a full-field digital mammography image. The recombined images (iodine map), as MRI, can reveal normal breast perfusion and lesions with higher vascularity and extracellular leakage of contrast (Fig. 3).

Low-energy images are acquired at a dose similar to a normal digital mammogram, while high-energy images have only about 20% of the dose of a normal digital mammogram [22]. Data regarding CEM radiation dose have been seldom reported [22]. In 11 studies considered for meta-analysis, the per-view average glandular dose of CEM ranged from 0.43 to 2.65 mGy [22]. At our institution, we use automatic regulation of CEM exposure parameters. In our viability assessment for CEM implementation in NAT setting, we recorded a mean average glandular dose of 2.4 mGy for the craniocaudal view and 2.7 mGy for the mediolateral oblique view, for a mean breast thickness of 5.3 cm for the craniocaudal view and of 5.8 cm for the mediolateral oblique view [26], which is well within the acceptable limit for mammography (2.5–3 mGy for 5.3–6.0 cm breast thickness) [27]. The patients’ summary of this analysis regarding patients’ radiation exposure using CEM in the NAT setting is detailed in supplementary materials (Table S1). In the NAT setting, the added radiation dose of CEM is of no significant biological consequence, because the patients will subsequently undergo either breast radiotherapy (which involves exposure to a much higher radiation dose) or mastectomy (which removes the exposed tissue).

After CEM, we monitor the patient while preserving the intravenous access for at least 15 min, to promptly recognize and treat any adverse reaction. Both iodinated and gadolinium contrast agents are associated with a very low rate of adverse effects [28]. The rate of adverse effects after intravenous iodinated contrast (0.15%) is higher than that of gadolinium (0.04%) [28]. In our experience, most adverse effects after CEM in the NAT setting are mild and managed at the Radiology Department [15, 18]. MRI requires exposure to intravenous gadolinium. There is clear evidence that the administration of various gadolinium-based contrast agents results in the accumulation of residual gadolinium in the brain and bones of patients, even in those with normal renal function [29]. Although the clinical significance of this scientific fact remains uncertain, it should be considered when choosing between non-inferior techniques that require repeated exams/exposures.

## NAT monitoring

Although nearly 80% of patients respond to NAT, only 6%–35% of patients achieve pathologic complete response (pCR), and approximately 5%–20% of patients show disease progression while receiving NAT [1–4]. Therefore, the need for accurate methods for monitoring NAT is well recognized. Indeed, it is an important part of the workload in everyday oncology clinical practice.

Presently, there is no established universal guideline regarding the optimal technique and time-points for imaging evaluation of NAT patients. National Comprehensive Cancer Network guidelines state that the multidisciplinary team of each institution should reach consensus, pointing out the need to consider the experience and techniques’ availability of each institution [30].

**Fig. 4:**
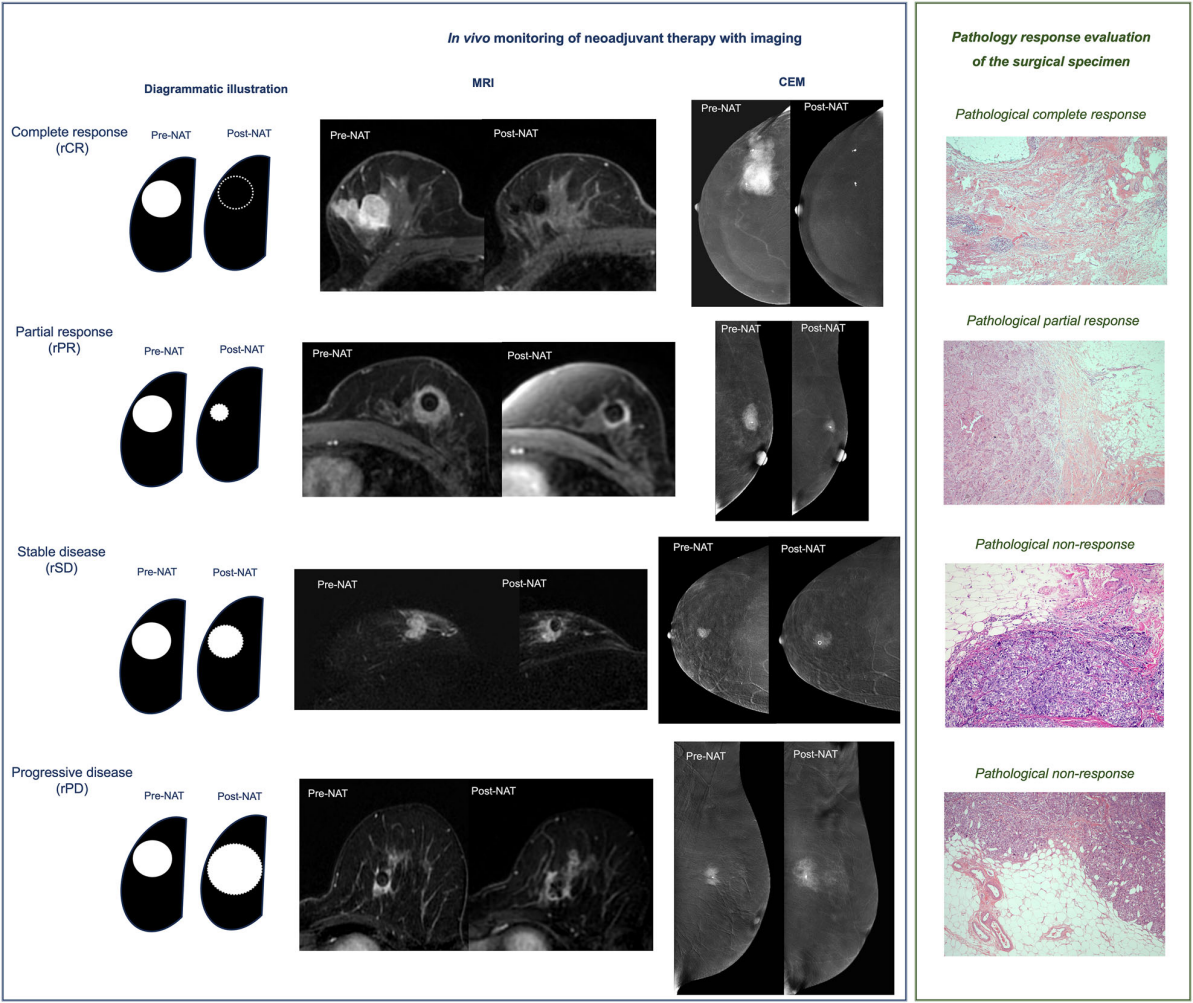
Correlation between NAT response assessment in vivo with advanced imaging techniques based on perfusion (CEM and MRI) and pathology response evaluation of the surgical specimen, according to American Joint Committee on Cancer (MRI: axial T1-weighted fat-suppressed image after intravenous gadolinium, CEM: recombined images). Complete response at imaging (rCR) is defined as the complete disappearance of the enhancing tumor. Complete response at pathology (pCR) is defined as the absence of invasive residual carcinoma in the breast and axillary lymph nodes (ypT0/is ypN0). Partial response at imaging (rPR) is defined as the reduction of at least 30% of enhancing target lesions. Partial response at pathology (pPR) is defined by the presence of invasive carcinoma with stromal alterations induced by NAT. Stable disease at imaging (rSD) is defined as an increase of less than 20% and a reduction of less than 30% of the enhancing target lesion. Progressive disease at imaging (rPD) is defined as the appearance of new lesions or an increase of at least 20% of enhancing target lesions. Both stable disease (rSD) and progressive disease (rPD) at imaging correspond to non-response at pathology, defined as the presence of invasive residual carcinoma without stromal alterations induced by therapy

NAT response evaluation is based on clinical and radiological data. Several studies have shown that contrast-enhanced MRI is superior to physical examination, mammography, and ultrasound to assess the extent of residual disease [6, 7]. This superiority has been mainly associated with the ability of MRI to distinguish fibrous from vascularized tissue after intravenous contrast administration. Like MRI, CEM evaluates both morphology and perfusion and, thus, it is a potential alternative for in vivo imaging assessment of NAT response (Fig. 4).

Pathology assessment of the surgical specimen is presently decisive for staging after NAT and is the reference standard for evaluating the performance of imaging techniques. Alike imaging, there are no universally accepted response assessment criteria for pathology. The main source of discrepancy among studies is whether to classify ductal carcinoma in situ (DCIS) as pCR or not. In a recent meta-analysis regarding imaging NAT assessment with CEM and/or MRI, 22 studies detailed the definition of pCR [13]. Ten of these 22 studies defined pCR as the absence of residual invasive cancer with or without DCIS in the completely resected breast specimen and all sampled regional lymph nodes (ypT0/Tis ypN0) as preconized by the American Joint Committee on Cancer [13, 31]. On the other hand, 12 studies considered that pCR required the absence of invasive cancer and of DCIS in the completely resected breast specimen and all sampled regional lymph nodes (ypT0 ypN0) [13]. The American Joint Committee on Cancer pathology response system (yAJCC) is a simple tool and a useful clinical prognostic marker for recurrence-free survival. yAJCC can directly correlate to the clinical TNM stage (cTNM), is widely accepted and used in clinical routine worldwide, and in our institution [32–34] (Fig. 4).

Residual Cancer Burden is another increasingly used and accepted system for reporting the extent of residual breast cancer after NAT. The Residual Cancer Burden score provides both a continuous (numerical) and categorical output and combines the extent of the remaining tumor in the breast and the axillary lymph nodes with other relevant prognostic factors, such as the amount of cellularity and size of nodal deposits [32, 33]. Both Residual Cancer Burden and yAJCC stage help predict recurrence [32–34]. Notably, in the I-SPY1 Trial, yAJCC and Residual Cancer Burden were non-concordant in 34% of patients, suggesting there is value in reporting both [34]. Indeed, at many institutions, pathologists are starting to routinely report both the yAJCC stage and the Residual Cancer Burden index and class [33, 34].

The method of evaluating treatment response with imaging has been the subject of many publications. Response Evaluation Criteria in Solid Tumors (RECIST) is widely used to standardize the radiological assessment of response to therapy of solid tumors [35, 36]. MRI is considered the standard imaging technique for RECIST assessment in the breast during NAT [35, 36]. The agreement of RECIST 1.1 response categories between CEM and MRI has been reported to be substantial at mid-NAT (κ = 0.791; 95% CI: 0.635–0.948) and almost perfect at post-NAT (κ = 0.871; 95% CI: 0.749–0.993) [11]. Figure 4 summarizes the correlation of NAT response assessment at advanced imaging (CEM and MRI) according to RECIST and at pathology according to yAJCC. Please note that stable disease (rSD) and progressive disease (rPD) on imaging are categories used for in vivo monitoring and that both are indistinguishable at pathology assessment, both corresponding to pathological non-response.

### CEM—the imaging alternative with increasing interest

Several publications have reported good initial experience with the use of CEM for NAT response assessment in breast cancer patients [8–15]. The comparison among studies is somewhat hampered by differences in study design quality, imaging evaluation, pathology analysis, definition of pCR, and systemic regimens evaluated [13, 14]. Table 1 resumes the studies that have directly compared the diagnostic performance of CEM and contrast-enhanced MRI for response evaluation of NAT and provide unequivocal, and FDA approved definitions of pCR.

**Table 1:** Head-to-head comparison of the diagnostic performance of CEM and contrast-enhanced MRI in the NAT setting

						CEM		MRI	
Authors (year) [reference]	Design	No. of patients	Systemic therapy	Definition of pCR	pCR (%)	Sensitivity % (95% CI)	Specificity % (95% CI)	Sensitivity % (95% CI)	Specificity % (95% CI)
Iotti et al [8]	Prospective	46	NAT	ypT0 ypN0	15	100 (63–100)	61 (43–76)	88 (47–100)	84 (69–94)
Patel et al [9]	Retrospective	65	NAT and endocrine therapy	ypT0 ypN0	31	95 (75–100)	67 (51–80)	95 (75–100)	69 (53–82)
Barra et al [10]	Prospective	33	NAT	ypT0/Tis ypN0	24	76 (55–91)	88 (47–100)	92 (74–99)	75 (35–97)
Bernardi et al [11]	Prospective	51	NAT	ypT0 ypN0	31	81 (54–96)	83 (66–93)	100 (79–100)	86 (70–95)
Hogan et al [12]	Prospective	110	NAT	ypT0 ypN0	28	94 (79–99)	64 (53–74)	69 (50–84)	73 (63–83)
Acar et al [16]	Prospective	74	NAT	ypT0/Tis ypN0	31.1	91.3 (72–98.9)	70.6 (56.2–82.5)	73.9 (51.6–89.8)	73 (60.4–85.7)

*pCR* pathologic complete response, *NAT* neoadjuvant therapy, *CEM* contrast-enhanced mammography

Two meta-analyses have reported CEM to be equivalent to contrast-enhanced MRI for NAT response assessment [13, 14]. In the most recent meta-analysis [14], the pooled sensitivity and specificity reported were 93% (95% CI: 84–97%) and 68% (95% CI: 60–76%) for CEM vs 84% (95% CI: 62–95%) and 80% (95% CI: 71–87%) for MRI. The AUC was 0.85 (95% CI: 0.82–0.88) for CEM and 0.85 (95% CI: 0.82–0.88) for MRI. These publications encourage the increasing use of CEM for NAT response assessment, although reporting an initial and limited experience.

### Pearls when monitoring NAT with CEM

**Fig. 5:**
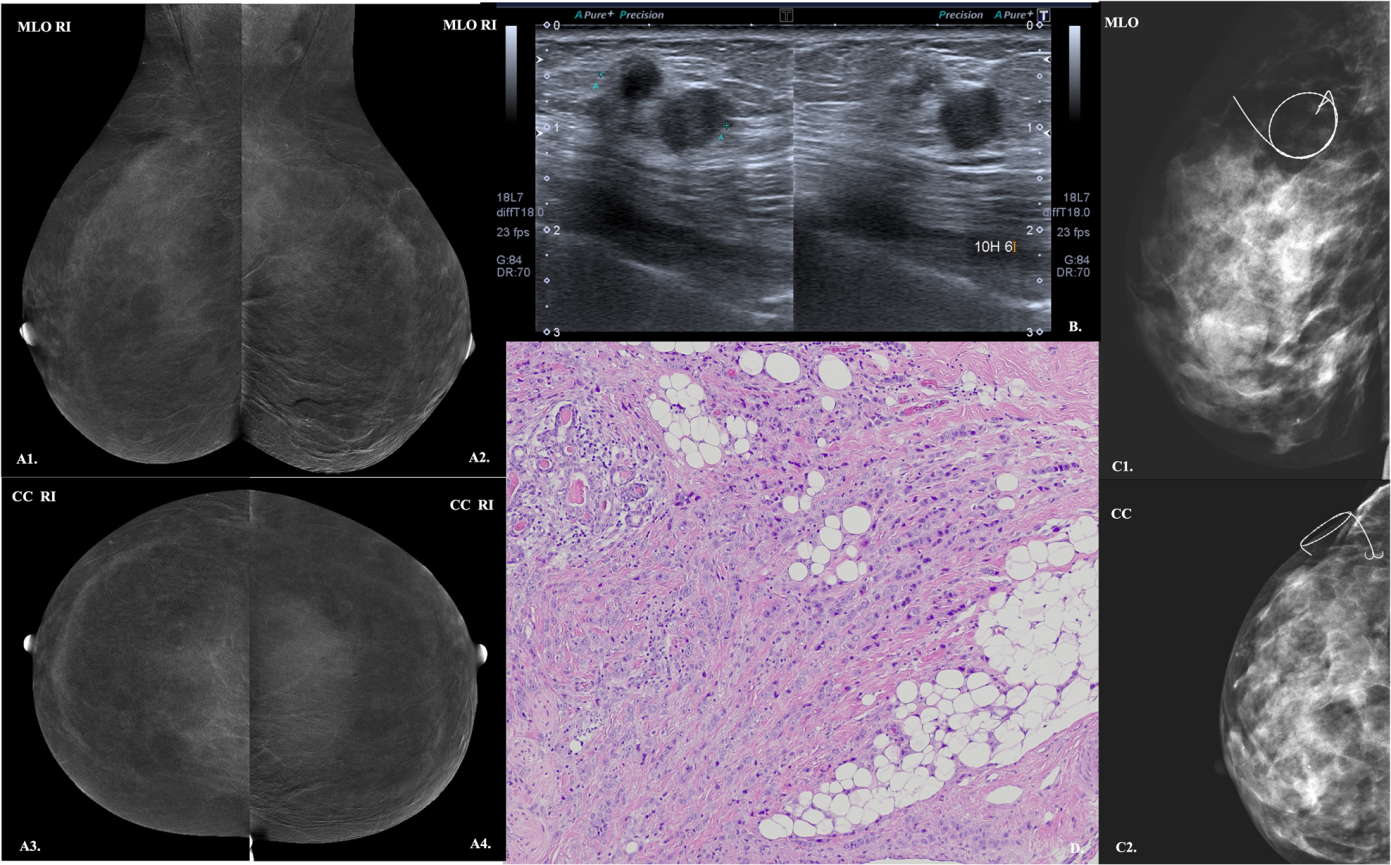
CEM false negative. CEM and ultrasound-guided core needle biopsy were performed in a 47-year-old woman presenting with a lump in the upper-outer quadrant (UOQ) of the right breast. This patient was not a candidate for NAT. **A** In the recombined images (iodine map) of CEM, no finding is demonstrated. **B** At ultrasound, in the UOQ and corresponding to a hard nodule at palpation, a nodule predominantly solid and slightly hypoechoic, oval, and with indistinct borders, is shown. Ultrasound-guided core needle biopsy was performed. The histology of the core needle biopsy demonstrates invasive breast carcinoma, with ductal and lobular features, grade 2, positive for estrogen and progesterone receptors, negative for human epithelial growth factor 2 receptors, and with a low Ki67 (< 15%). **C** The mammography performed after wire-guided marking of the lesion, demonstrates its mammographic location, where no mammographic findings are shown. **D** The histology of the surgical piece confirmed invasive carcinoma, with ductal and lobular features, grade 2, “luminal A” like type, with non-extensive DCIS, of intermediate to high nuclear grade, measuring 14 mm. The surgical margins were adequate, and the sentinel axillary lymph nodes were negative. The tumor was staged as pT1cN0(sn). The patient was treated with breast radiotherapy and hormone adjuvant therapy and is disease-free in the seventh year of follow-up. MLO, mediolateral oblique view; CC, craniocaudal view; LE, low energy; RI, recombined image

The use of CEM for NAT response assessment requires a baseline study (before starting NAT) and at least an additional CEM after the end of NAT for final response assessment and adequate surgical planning. The baseline study evaluates tumoral visibility at CEM. The false-negative rate for cancer has been estimated to be 4%–9% for CEM [23]. At our institution, we have cases in which the tumor did not enhance at CEM (Fig. 5). In these patients, when NAT is indicated, CEM cannot be used for imaging response assessment.

A baseline bilateral CEM completes initial staging and evaluates background parenchymal enhancement. The amount of background parenchymal enhancement at CEM may be described as symmetric or asymmetric and as minimal, mild, moderate, or marked, similarly to MRI [37]. It is important to remember that CEM is a planar exam and that enhancing lesions may blend with background parenchymal enhancement when it is moderate or marked. Indeed, this baseline bilateral approach is particularly helpful in infiltrative and diffuse multicentric breast cancer (Fig. 3). Thereafter, CEM may be restricted to the diseased breast, to minimize radiation exposure. It should be noted that inflammatory breast cancer is considered a non-measurable lesion according to RECIST 1.1 guidelines [35].

Bernardi et al reported that a delayed CEM mediolateral view acquisition (6 min after injection of contrast) could detect additional cases of residual DCIS after NAT and had higher specificity for pCR in comparison with CEM without a delayed phase [11]. The rationale for adding a delayed CEM view would be to better depict the diffusion of iodinated contrast, which can be slowed by the anti-vascular effect of NAT. Yet the performance of CEM for NAT response assessment observed with and without delayed CEM exhibits no statistically significant difference (sensitivity: CEM 81% vs CEM with delayed view 81%, *p* > 0.99; specificity: CEM 83% vs CEM with delayed view 89%, *p* > 0.41) [11].

A limitation of CEM is a more restricted anatomic coverage compared to MRI. Indeed, MRI’s larger field of view can depict the totality of peripherally located breast tumors, as well as mammography blind spots, including the chest wall, axilla, and internal mammary lymph nodes. Patients’ weight loss and tumor reduction, often induced by NAT, may further exacerbate it and have been described as a cause of drop-off for CEM’s response assessment to NAT [15, 18]. This observation underlines the need to carefully consider these possible effects of NAT in patients’ initial imaging selection, particularly in the setting of posterior/peripherally located tumors or in patients with smaller breast cups.

Breast-conserving surgery after NAT requires marking the cancer site with a metallic marker. All metallic markers have no significant artifact in the surrounding tissues at mammography and minimal artifact in recombined CEM images. Compared with MRI, mammography allows a more straightforward confirmation of the location of the intra-mammary metallic marker. At MRI, the same susceptibility artifact (signal void) that accounts for the visibility of the marker may hinder NAT response assessment. There are considerable variations in the artifact size and characteristics across different clips, MRI sequences, and field strengths [38]. Particularly, magnetic seeds may create a bloom artifact at MRI, which can measure up to 4 cm. Indeed, CEM has been proposed as a suitable alternative for NAT monitoring when magnetic seeds are placed [39].

**Fig. 6:**
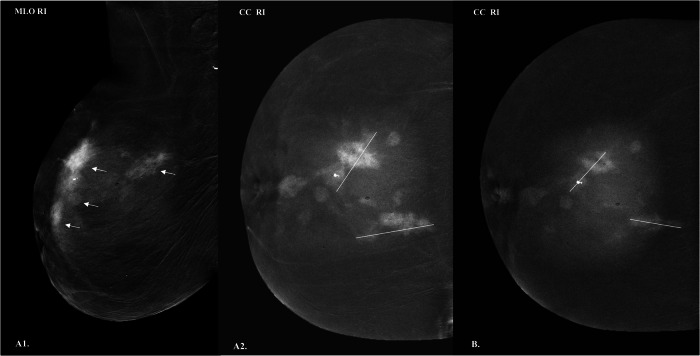
Radiologic interpretation of CEM for neoadjuvant treatment (NAT) monitoring, in a 66-year-old patient with an invasive breast carcinoma of no special type (NST), with positive estrogen and progesterone receptors, and negative human epithelial growth factor 2 receptors (HER2), consistent with “luminal” like molecular subtype. **A1** In the CEM mediolateral oblique view of the left breast, at baseline, some of the tumoral lesions are superimposed (arrows). **A2** CEM craniocaudal view, at baseline, was found to better depict the lesions and was chosen for comparative analysis. **B** After NAT, a decrease of 34% of the longest dimension was shown, consistent with radiological partial response and confirmed as pathological partial response in the surgical specimen. MLO, mediolateral oblique view; CC, craniocaudal view; RI, recombined image

In clinical practice, the measurement changes used to predict response follow the RECIST 1.1 criteria (Fig. 4) [35, 36]. Because CEM is a planar technique that requires two views, it is important to choose the view that better depicts the lesions individually and allows comparative measurements (Fig. 6). The same methodology should be used for CEM comparison and measurement of the longest dimension of the target lesion(s) in all time points. In cases of multiple neoplastic lesions at baseline, a maximum of five target lesions or, as the breast is a paired organ, two target lesions per breast, can be chosen for the measurement change assessment (Fig. 6) [35, 36, 40]. The remaining enhancing lesions in the breast are considered non-target lesions. Therefore, they should be evaluated at follow-up imaging, but are not required to be measured.

### Pitfalls when monitoring NAT with CEM

Benign enhancing breast lesions may also exhibit decreased enhancement after NAT at CEM or at MRI [41, 42]. NAT may induce nonspecific changes mediated by antiangiogenic effects associated with taxanes and ovarian failure, most closely associated with alkylating agents (e.g., cyclophosphamide), which affect breast cancer and benign breast lesions similarly. Indeed, size reduction and decreased enhancement of an enhancing breast lesion during NAT should not be misinterpreted as evidence of malignancy [41].

Since both CEM and MRI rely on perfusion, it is conceivable that both share the same rationale for radio-pathological discrepancy in the NAT scenario. Indeed, CEM and MRI have been reported to similarly predict the size of pathological residual disease after NAT [8, 11, 12], regardless of the definition of pCR adopted (for ypT0/Tis ypN0 *p* = 0.52 and for ypT0 ypN0 *p* > 0.99) [12]. In the study that specifically addressed this issue and had the largest sample, size, correlation with pathological residual disease was moderate for both CEM and MRI, and both imaging techniques tended to overestimate pathological lesion size [12].

**Fig. 7:**
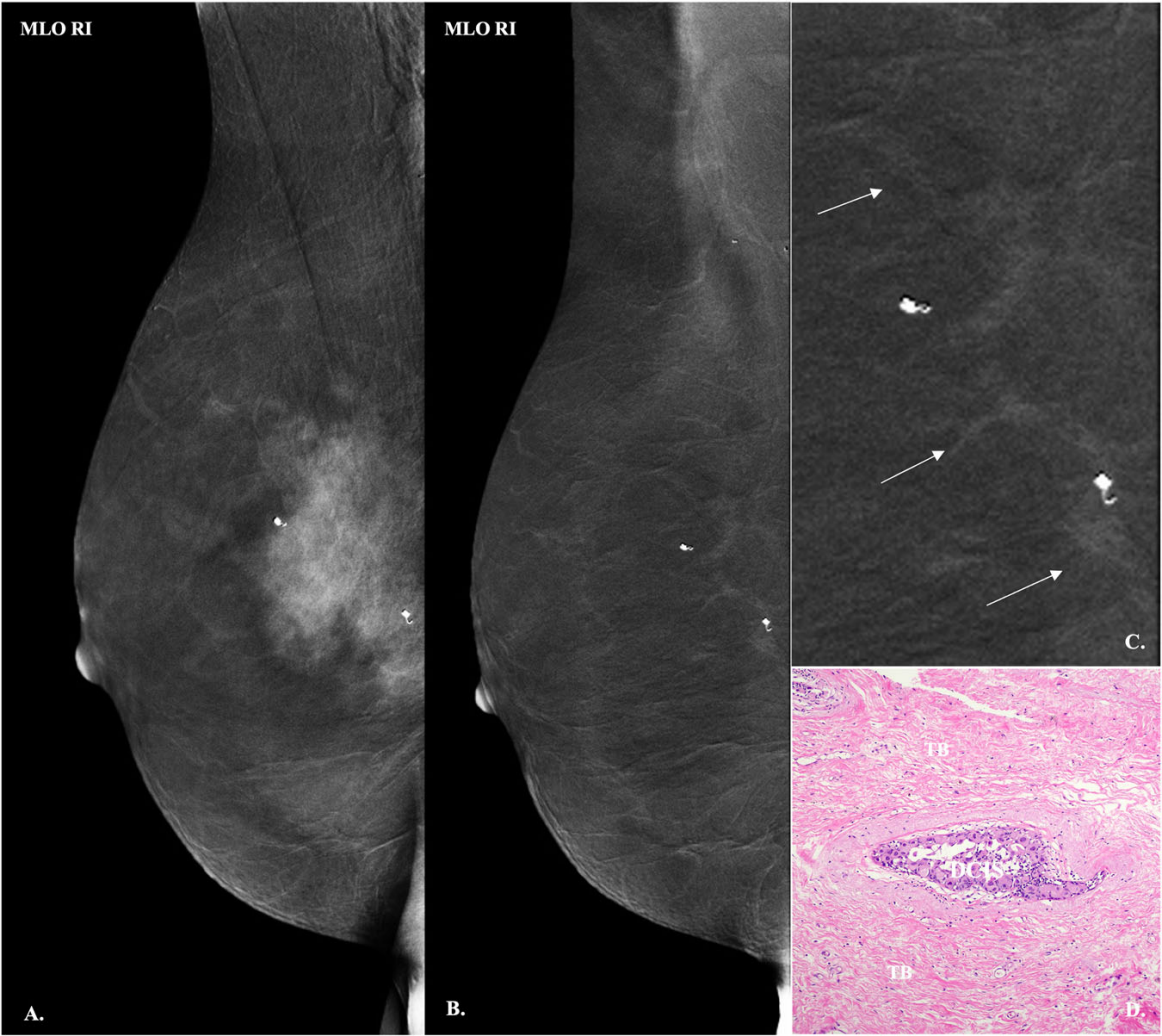
CEM response assessment of NAT in cancers associated with enhancing DCIS. **A** At baseline, CEM of the right breast, a non-mass enhancement, marked with two clips, is shown. The histology of the core needle biopsy before NAT demonstrated an invasive breast carcinoma of NST, grade 2, negative for estrogen, progesterone, and human epithelial growth factor 2 receptors (triple negative subtype), with coexisting DCIS of high nuclear grade. **B** At CEM after NAT, the tumoral area, marked with two clips, exhibits a smaller and less enhancing non-mass lesion, favoring radiological partial response. **C** The residual non-mass enhancing lesion is better displayed in the amplification of B (arrows), favoring radiological partial response. **D** The histology of the partial mastectomy demonstrates a fibrotic tumor bed, without residual invasive carcinoma (marked as TB in **D**), with coexisting residual DCIS (marked as DCIS in **D**), of high nuclear grade, measuring 30 mm, consistent with pathological complete response (pCR) and in perfect correlation with the finding displayed at CEM after NAT. MLO, mediolateral oblique view; RI, recombined image

**Fig. 8:**
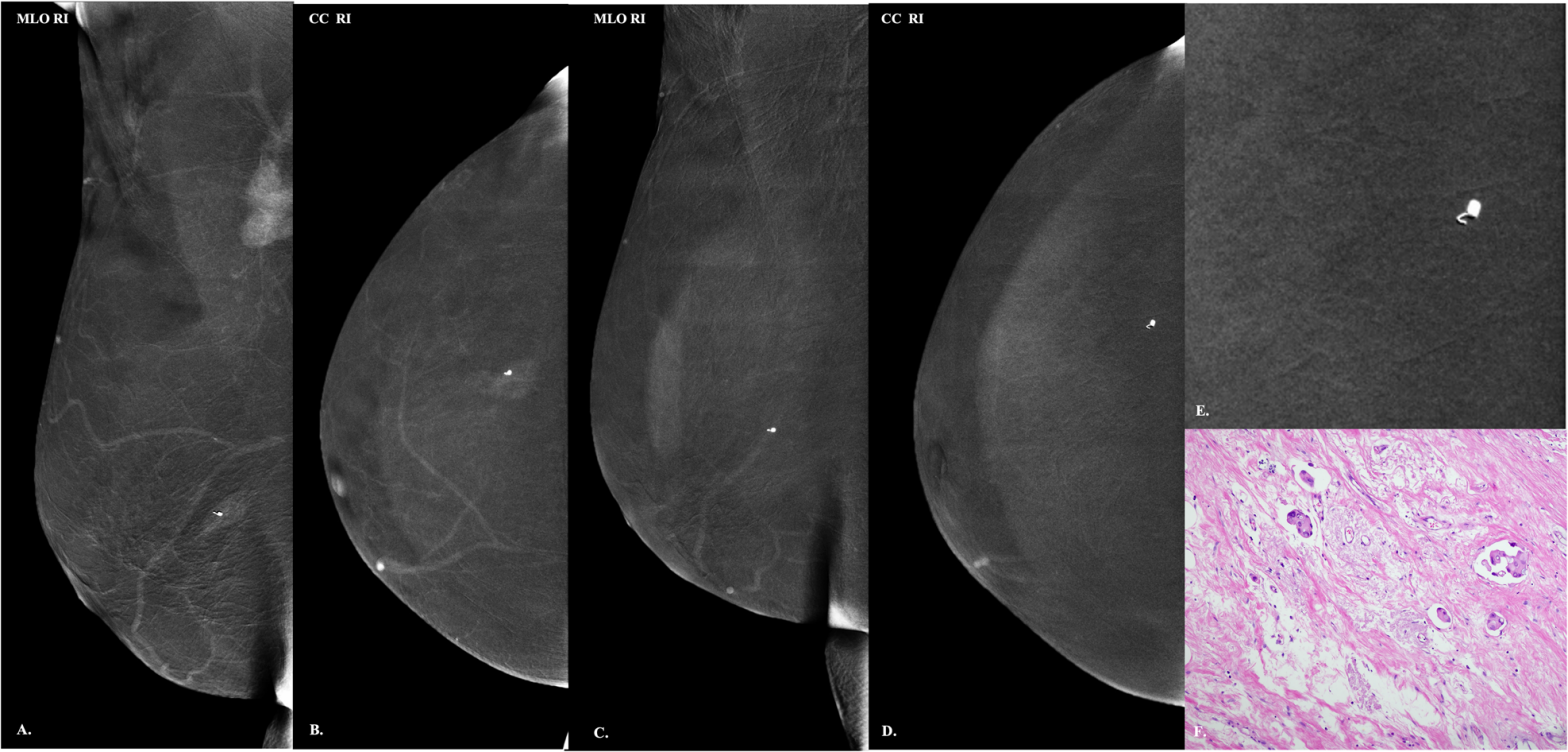
CEM response assessment of NAT in cancers of the luminal molecular subtype and weak enhancement. **A**, **B** At baseline CEM of the right breast, a low to moderate enhancing mass, marked with a clip, is shown. The histology of the core needle biopsy before NAT demonstrated an invasive breast carcinoma of NST, grade 2, with positive estrogen and progesterone receptors, and negative human epithelial growth factor 2 receptors (HER2), consistent with “luminal” like molecular subtype. Notice also the enhancing axillary adenopathy in A, only partially covered at CEM. **C**, **D** At CEM, after NAT, the tumoral area, marked with a clip, exhibits no residual enhancement. **E** The absence of residual enhancement is better displayed in the amplification of D, favoring radiological complete response. **F** The histology of the partial mastectomy demonstrated a tumor bed with residual invasive carcinoma NST, grade 3, predominantly of low cellularity, measuring 25 mm, consistent with a pathological partial response. MLO, mediolateral oblique view; CC, craniocaudal view; LE, low energy; RI, recombined image

**Fig. 9:**
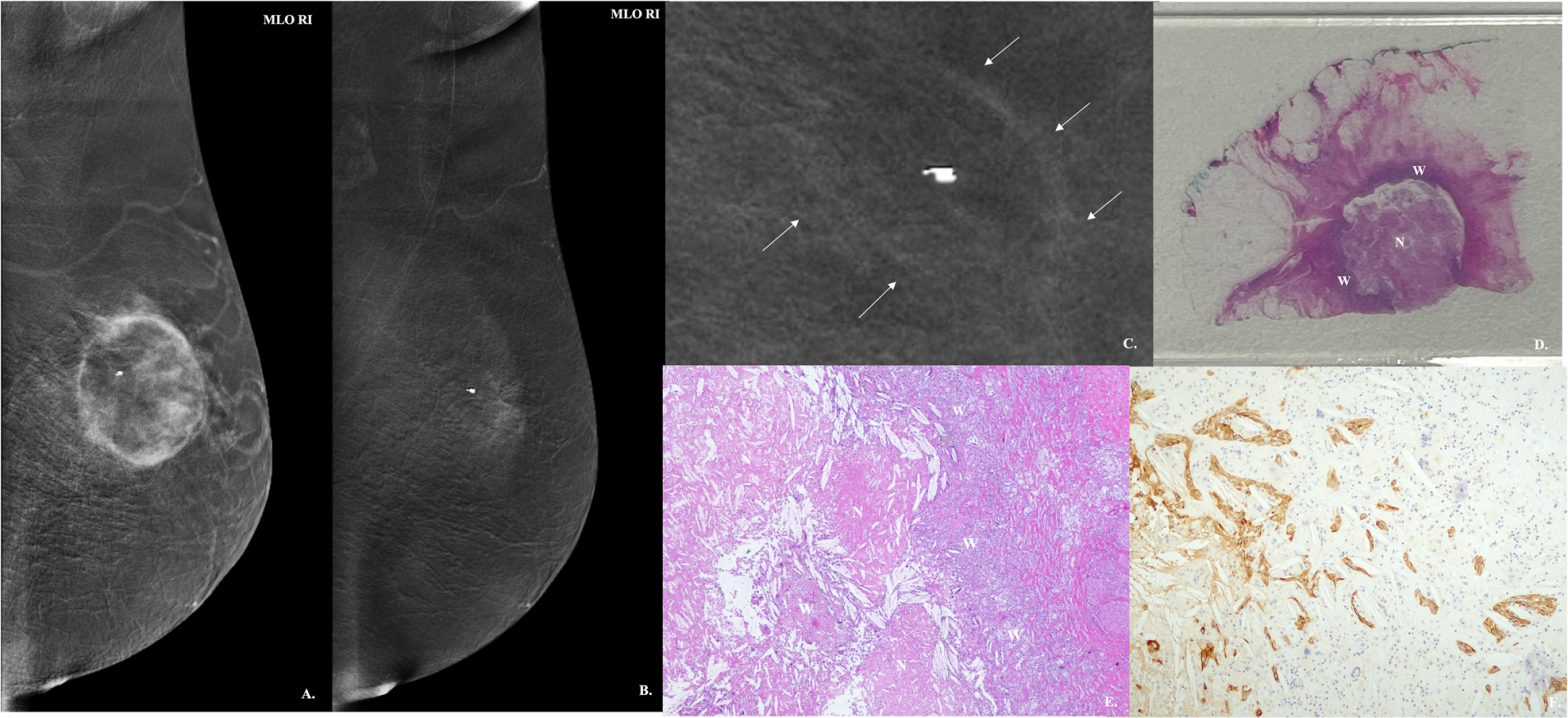
CEM response assessment of NAT in cystic cancers. **A** At baseline CEM of the left breast, a rim-enhancing mass with heterogeneous internal enhancement, marked with a clip, is shown. The histology of the core needle biopsy before NAT demonstrated an invasive carcinoma with squamous (metaplastic) features, grade 3, and weak estrogen receptor positivity (10%–20%), progesterone receptor negativity, and human epithelial growth factor 2 receptors (HER2) positivity at fluorescence in situ hybridization, consistent with HER2-positive molecular subtype. **B** At CEM after NAT, the cancer exhibits size and enhancement reduction with a peripheral and irregular rim and internal septal weak enhancement. **C** The residual enhancement is better displayed in the magnification of (**B**). (arrows), consistent with radiological partial response. **D** At pathology gross inspection of the surgical specimen, the residual tumor corresponds to a cystic lesion with a necrotic center and an irregular wall, suspicious for invasion of the surrounding breast parenchyma, in perfect correlation with the CEM findings. **E**, **F** The histology of the tumor bed consisted of a cystic area with an irregular wall (marked as W in **D**, **E**) surrounding a necrotic center with inflammatory debris (marked as N in **D**, **E**). In the periphery of the wall, small foci of residual invasive carcinoma were found (positive marking for cytokeratin AE1/3 in **F**), consistent with pathological partial response. MLO, mediolateral oblique view; RI, recombined image

Studies regarding MRI evaluation for NAT response have found that 6%–19% of cases are overestimated and 7%–28% of cases are underestimated [40]. Overestimation of residual disease after NAT at MRI may be related to enhancing residual DCIS if pCR is defined as ypT0/Tis ypN0 (Fig. 7), chemotherapy-induced fibrosis or reactive inflammation associated with tumor response and healing [6, 7, 14, 40]. Possible reasons for underestimation in this setting include: residual invasive cancer at the tumoral bed, only present as very small foci of residual cancer (< 0.1 cm) in the/near the limit of visibility, weak enhancing tumors at baseline (Fig. 8), luminal molecular subtype (Fig. 8), low histological grade, non-mass enhancement, infiltrative lesions, cystic carcinomas (Fig. 9), invasive lobular carcinoma, non-concentric shrinkage pattern and multicentricity [6, 7, 14, 40]. Nonconcentric shrinkage, also called crumbling, fragmented, or multinodular pattern, corresponds to a shrinkage pattern in which a single lesion at baseline responds to NAT by crumbling into several lesions [40]. It corresponds to a partial response. Residual disease in this shrinkage pattern is more difficult to ascertain. Enhancing residual disease can be interspersed with enhancing areas of reactive fibrosis or of DCIS, therefore, increasing the likelihood of both over- and underestimation [40].

One of the NAT purposes is to downstage the regional lymph node metastasis, with reported pCR rates of 35%–68% for metastatic axillary lymph nodes [40]. A CEM major limitation is its restricted field of view. Indeed, the mediolateral oblique view of CEM can only cover a limited portion of the level I axilla (Fig. 8). Presently, the accurate evaluation of axillary lymph node metastasis response to NAT relies on the pathology assessment of the surgical specimen, obtain through sentinel lymph node biopsy (false negative rate 5.9%), targeted axillary dissection (false negative rate 2%) or axillary lymph node dissection, as indicated by the imaging predicted and biopsy-proven metastatic regional lymph node burden [40, 43]. Indeed, a meta-analysis reported that the diagnostic performance of imaging techniques for the assessment of axillary metastatic disease after NAT was limited, with a false negative rate of 35% for ultrasound, of 40% for MRI, and of 62% for 18F-FDG positron emission tomography/computed tomography (PET-CT) [44]. According to the American College of Radiology Appropriateness Criteria, ultrasound is the most appropriate imaging technique for the assessment of residual disease in the axillary lymph nodes after NAT [45] and could complement CEM in response monitoring during NAT and preoperative staging.

### CEM in the setting of NAT: opportunities and challenges

#### From the oncologist's perspective

**Fig. 10:**
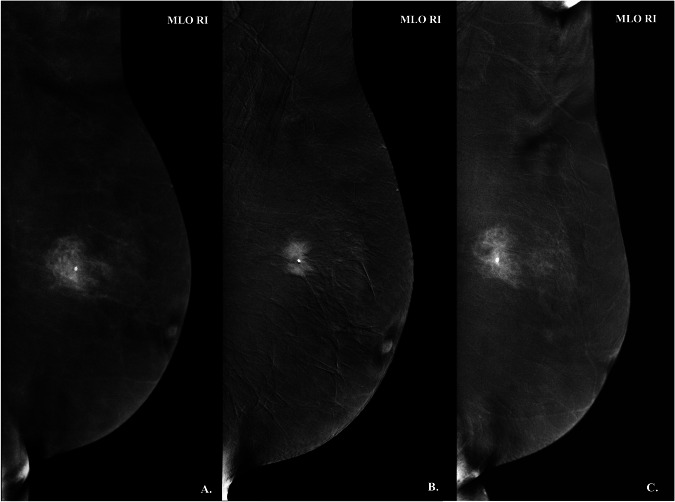
CEM in vivo monitoring of response to NAT in a 43-year-old patient with an invasive breast carcinoma of NST, grade 3, of the triple negative molecular subtype (negative for estrogen, progesterone, and human epithelial growth factor 2 receptors). **A** Pre-NAT CEM of the left breast demonstrates an enhancing mass, marked with a clip, corresponding to the cancer. **B** At mid-NAT, after completing the first type of NAT regimen (paclitaxel and carboplatin), a radiological partial response is shown. **C** After completing the second type of NAT regimen (doxorubicin and cyclophosphamide dose dense), radiological progression is demonstrated, confirmed as pathological non-response, in the histology of the mastectomy and axillary lymph node dissection specimens. MLO, mediolateral oblique view; RI, recombined image

NAT presents a unique opportunity to test chemosensitivity in vivo. Radiological monitoring during NAT can dynamically monitor the cancer evolution and its degree of response to a specific NAT regimen (Fig. 10). Currently, in clinical practice, the information of radiological response is used to exclude progression during NAT and to achieve preoperative staging. Approximately 5%–20% of patients exhibit disease progression while receiving NAT [1–4], which must prompt a change of treatment, such as switching systemic therapies and/or anticipating surgery (Fig. 10).

Pathological response is the cornerstone for the prediction of survival, assisting in the decision of adjuvant treatments after NAT. Although pathological response after NAT is an established prognostic marker, it cannot be used as an early predictor.

Improvements in systemic therapy and the availability of targeted therapies have greatly increased the rates of pCR. Unfortunately, this conquest has led to increased costs and risk of toxicity. There is growing interest in the de-escalation of NAT and in treatments tailored to in vivo response [46]. The early stratification of response could allow for patient-centered choice of NAT regimens, optimizing the trade-off between therapeutic gains and iatrogenic hazards, in both responders and non-responders. This could spare patients and society the physical, psychological, and financial burden of potentially ineffective and toxic treatments [15, 46].

Indeed, with increasing use of precision cancer medicine, the need for early predictors of response has gain ever more relevance. Imaging allows early and in vivo monitoring of cancers. Advanced morphofunctional imaging techniques that reflect tumor biology and in vivo response, such as MRI, PET-CT, and CEM, have been explored as early indicators of response to NAT in breast cancer patients with encouraging results [15, 46]. CEM has a logistical profile superior to MRI and PET-CT. Indeed, CEM’s low cost, short examination time, good tolerance, and potential high availability make it an ideal technique for performing repeated evaluation required for in vivo response monitoring [15, 18].

**Fig. 11:**
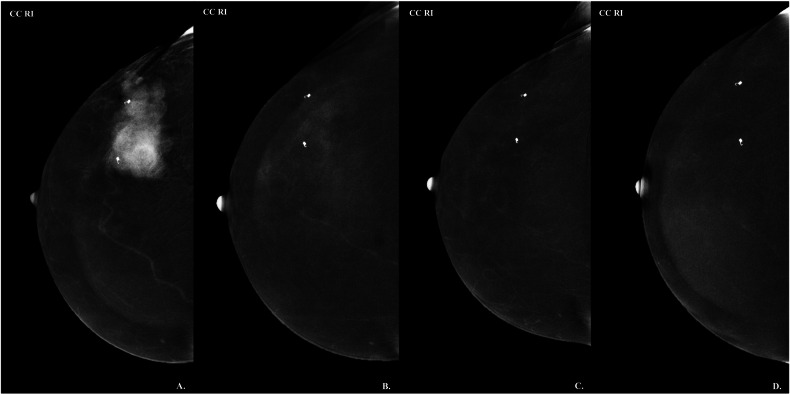
CEM early prediction of response to NAT in a 52-year-old patient with an invasive breast carcinoma of NST, grade 3, of the triple negative molecular subtype (negative for estrogen, progesterone, and human epithelial growth factor 2 receptors). **A** Pre-NAT CEM of the right breast demonstrates an enhancing mass, marked with two clips, corresponding to the cancer. **B** After completing the first cycle of NAT, the cancer exhibits a percentage change in the longest dimension of the lesion (CLD) superior to 21% at CEM, predictive of pCR. The CLD is calculated using the following formula: (longest dimension of the lesion before NAT—longest dimension of the lesion after the first cycle of NAT)/longest dimension of the lesion before NAT. **C** At mid-NAT, after completing the first type of NAT regimen (paclitaxel and carboplatin), radiological partial response is shown. **C** After completing the second type of NAT regimen (doxorubicin and cyclophosphamide dose dense), a radiological complete response is demonstrated, confirmed as pCR, in the histology of the partial mastectomy and of the sentinel axillary lymph node specimens. CC, craniocaudal view; LE, low energy; RI, recombined image

An earlier investigation obtained initial encouraging results with CEM as an early predictor of response (Fig. 11). CEM-derived measurement changes after the first cycle of NAT were an independent predictor of pCR after NAT (OR, 9.52; *p* = 0.02), with good negative predictive value (88%) and substantial inter-reader agreement (κ = 0.76) [15]. Interestingly, among participants with hormone receptor-negative breast cancer (triple-negative and HER2-positive molecular subtypes), acknowledged as good responders to NAT, early changes in CEM-derived measurements were associated with a greater likelihood of pCR (OR, 40.00; *p* = 0.005) [15]. Although promising, these early results should be viewed with caution due to the small sample size, and larger multi-center studies are needed to clarify the role of CEM as an early predictor of pCR.

#### From the surgeon's perspective

Tumoral size reduction is one of the main goals of NAT. The improved surgical cosmesis and reduced morbidity of breast and axilla preserving surgery, must be weighed against the possibility of inadequate surgical margins. Positive resection margins lead to re-surgery, to avoid the increased long-term risk of disease recurrence. Although patients with residual DCIS only have a better prognosis than patients with residual invasive disease, the absence of both DCIS and invasive disease has superior disease-free survival than residual DCIS only [12].

**Fig. 12:**
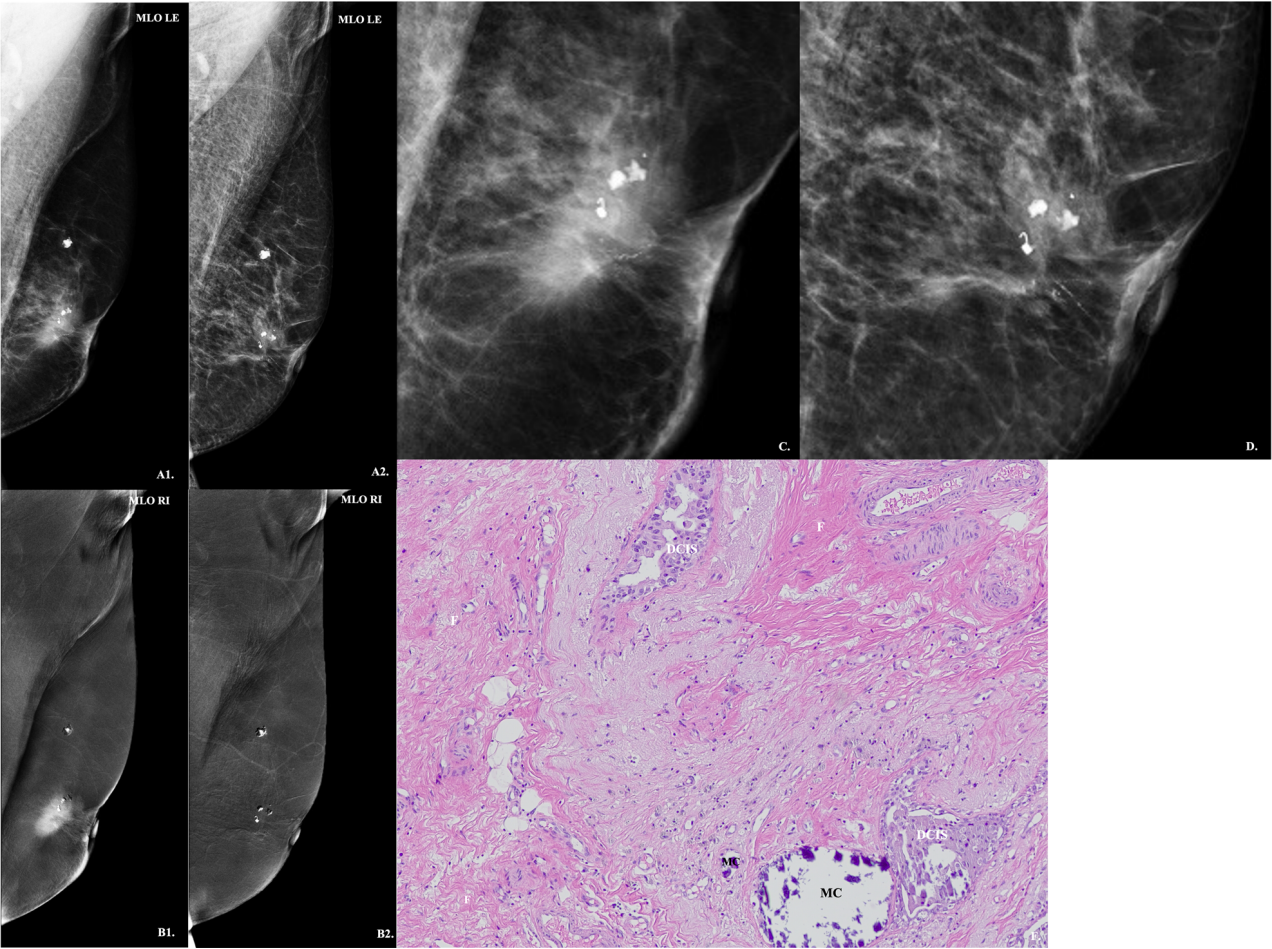
CEM preoperatory assessment of invasive breast carcinoma, of the human epithelial growth factor 2 receptors (HER2) positive molecular subtype (positive for estrogen, progesterone, and human epithelial growth factor 2 receptors), with coexistent DCIS after NAT. **A1**, **B1** Pre-NAT CEM of the left breast demonstrates an enhancing mass, marked with a clip, and associated nipple retraction and fine pleomorphic microcalcifications. The histology of the core needle biopsy before NAT demonstrated an invasive breast carcinoma of NST, with micropapillary features, grade 2, and coexisting DCIS of intermediate to high nuclear grade. **A2**, **B2** Post-NAT CEM displays complete disappearance of the enhancing mass, marked with a clip, as well as partial resolution of nipple retraction, favoring radiological complete response. **C**, **D** The magnifications of A1 (**C**) and A2 (**D**) better depict an increase in the extent of the fine pleomorphic microcalcifications after NAT. **E** The histology of the partial mastectomy demonstrated fibrotic tumor bed without residual invasive cancer (marked as **F**), consistent with pCR, and residual DCIS (marked as DCIS), of intermediate to high nuclear grade, measuring 14 mm, with adequate margins. Notice also the presence of pleomorphic microcalcifications (marked as MC). MLO, mediolateral oblique view; LE, low energy; RI, recombined image

From the surgeon's perspective, accurate evaluation of both residual invasive carcinoma and DCIS after NAT are essential for surgical planning (Fig. 12). Identifying and mapping the totality of residual DCIS, regardless of pCR, and the presence and distribution of viable tumor, particularly if in the form of scattered foci, are of paramount importance to avoid repeated surgery.

Presently, the standard practice is to remove the totality of suspicious microcalcifications in the tumoral bed, as it is not possible to distinguish microcalcifications associated with viable DCIS and invasive cancer from those associated with necrotic tumor. Microcalcifications are only adequately depicted by mammography. Indeed, MRI plus mammography has been reported to be superior to MRI alone in accurately selecting patients for breast-conserving surgery after NAT [47]. CEM (low-energy images + recombined images) and breast MRI + low-energy images performances for detection of residual invasive cancer and DCIS have been reported to exhibit no significant difference (*p* > 0.99) [12]. Notably, CEM can evaluate both microcalcifications, and residual enhancement, allowing preoperative planning in a single study [12].

#### From the pathologist's perspective

Pathological assessment of post-NAT surgical specimens may be challenging. It requires systematic sampling of an adequate area of the breast and standardized measurements of size and cellularity of the invasive residual carcinoma in the breast and in the axillary lymph nodes [34, 48]. Post-NAT, the tumoral area may be difficult to identify at gross pathological examination, often being softer than pre-NAT or without suggestive findings [34, 48]. Tumor response is frequently heterogeneous, and residual disease frequently corresponds to scattered foci of invasive carcinoma in a fibrous tumor bed [34, 48]. When no residual carcinoma is present, extensive sampling may be required. Indeed, an accurate methodology is of utmost importance to document the absence of something.

Multidisciplinary collaboration and correlation are essential for successful pathology assessment [34, 48]. All former location information (from communication with the surgeon, imaging, previous biopsy reports, and/or clip location) is used to identify the location of the residual tumor. Adequate pathology sampling of the correct area of the breast is more important than exhaustive sampling of any visible fibrotic area or than blindly submitting a required number of blocks. X-ray of the surgical specimen at the Radiology Department and/or surgical specimen dedicated X-ray machines available at the Pathology Department may be helpful to identify the tumor area, particularly when it has been marked with a clip or has coexisting microcalcifications. CEM may not only map the residual enhancing tumor, like MRI [8–14], but also depict clips and microcalcifications that mark the tumor area, like mammography [12, 48]. Indeed, CEM can map the totality of the tumoral area imaging identifiers and provides a more straightforward correlation with the X-ray of the surgical specimen, facilitating pathology assessment after NAT.

#### From the patient's perspective

During NAT, patients are exposed to frequent procedures and considerable side effects in a relatively short period of time. Hence, especially when repeated imaging is required, such as for NAT monitoring, acceptability to patients must be an important consideration when choosing any imaging technique. Compared to MRI, CEM is a preferred and better-tolerated exam. As shown by an earlier investigation, in the assumption of equal accuracy for NAT response assessment, most participants (76%) preferred CEM over MRI [18]. The preference for CEM was justified by the perception of shorter duration (*p* < 0.001), greater overall comfort (*p* < 0.01), more comfortable positioning (*p* = 0.01), lower anxiety (*p* = 0.03), and absence of noise (*n* = 15) [18]. It is reassuring that NAT patients find the additional burden of repeated CME examinations acceptable, on top of treatment, toxicity, and other diagnostic procedures [18].

#### From the logistical perspective

With the increasing use of NAT, the need for equitable access to appropriate high-quality advanced imaging gains momentum. The choice of CEM offers many possible advantages. It is faster and less expensive than MRI [17]. CEM has no associated risk of claustrophobia, is not limited by body habitus, does not require the prolonged prone position, which may preclude MRI, and is not contraindicated in patients with electronic or metallic devices. Indeed, it is consensual that CEM should be offered to patients when an MRI cannot be performed.

The potential high availability of CEM is particularly relevant in the setting of repeated imaging as required for NAT response assessment. Access to MRI may be limited even in industrialized countries, in which breast MRI competes with other imaging fields, such as musculoskeletal referrals. MRI exams are expensive and time-consuming, considering both the image acquisition and the radiologist's interpretation time. Our feasibility assessment for CEM implementation in the NAT setting included two readers with 7 years and 22 years of experience in multimodality breast imaging and two years of experience reading CEM [26]. The median reading time of CEM assessment of NAT response was short (median: 3.5 min; range: 1–15 min) and compared favorably with that of MRI (median: 6.6 min; range: 2–22 min) in the same reading setting (comparison of three examinations: baseline, at mid-NAT, and after NAT) and in the same cohort [26]. Compared to MRI, CEM requires a totally dedicated breast machine (with no competing agenda), has a lower implementation and maintenance cost, a fast-learning curve, and is a faster exam to perform and to read, allowing for greater patient flow rate. Indeed, CEM holds the promise of facilitating radiologists’ daily workloads and improving patient throughput, promoting equitable access to appropriate high-quality advanced imaging.

## Conclusion

The data compiled herein reflect a positive early experience, encouraging further development of CEM in the NAT setting, not only from the radiologist's view but also from a broad multidisciplinary perspective. Several knowledge gaps remain regarding CEM for NAT imaging assessment, particularly standardization of the patients’ selection process for monitoring NAT with CEM, CEM interaction with molecular biomarkers, such as liquid biopsy, and CEM interplay with alternative imaging techniques, such as PET-CT, or emerging tools, such as artificial intelligence, dedicated breast CT, and photon-counting CT.

## Supplementary information

ELECTRONIC SUPPLEMENTARY MATERIAL

## Data Availability

Available upon reasonable request.

## Abbreviations

CEM: Contrast-enhanced mammography
DCIS: Ductal carcinoma in situ
HER2: Human epidermal growth factor receptor 2
MRI: Magnetic resonance imaging
NAT: Neoadjuvant therapy
pCR: Pathologic complete response
PET-CT: Positron emission tomography/computed tomography
RECIST: Response Evaluation Criteria in Solid Tumors 1.1
yAJCC: American Joint Committee on Cancer Pathology Response System
