# Supplementary material for: Contrast-enhanced mammography in the setting of neoadjuvant therapy from a multidisciplinary perspective
Source: Insights Imaging. 2026 Jul 15;17:184. doi: 10.1186/s13244-026-02294-5 (PMC13373129; doi:10.1186/s13244-026-02294-5)
Supplement: Supplementary file 1 — ELECTRONIC SUPPLEMENTARY MATERIAL [file 13244_2026_2294_MOESM1_ESM.pdf]

Contrast-enhanced Mammography in the setting of Neoadjuvant Therapy from a Multidisciplinary Perspective

ELECTRONIC SUPPLEMENTARY MATERIAL

Insights Imaging (2026) Pires-Gonçalves L, Leal C, Guimarães-Santos A, et al.

Supplementary Table S1. Patients' summary regarding patients' radiation exposure using CEM in the NAT setting

Patient number	MGD_LE count (dGy)	MGDtotal Sum (dGy)
**1**	10	0,290099
**L**	8	0,239105
20180430	2	0,052801
CC	1	0,026704
MLO	1	0,026097
20180619	2	0,057223
CC	1	0,028575
MLO	1	0,028648
20180822	2	0,056962
CC	1	0,028631
MLO	1	0,028331
20181017	2	0,072119
CC	1	0,030806
MLO	1	0,041313
**R**	2	0,050994
20180430	2	0,050994
CC	1	0,029866
MLO	1	0,021128
**2**	8	0,235027
**L**	2	0,048649
20180430	2	0,048649
CC	1	0,020703
MLO	1	0,027946
**R**	6	0,186378
20180430	2	0,067071
CC	1	0,033531
MLO	1	0,03354
20180725	2	0,067198
CC	1	0,028124
MLO	1	0,039074
20181015	2	0,052109
CC	1	0,0209
MLO	1	0,031209
**3**	8	0,258124
**L**	6	0,192111
20180509	2	0,060947
CC	1	0,026018
MLO	1	0,034929
20180628	2	0,059263
CC	1	0,019765
MLO	1	0,039498
20181114	2	0,071901
CC	1	0,030785

MLO	1	0,041116
**R**	2	0,066013
20180509	2	0,066013
CC	1	0,022657
MLO	1	0,043356
**4**	10	0,341449
**L**	8	0,275341
20180509	2	0,073855
CC	1	0,038581
MLO	1	0,035274
20180620	2	0,073538
CC	1	0,037745
MLO	1	0,035793
20180913	2	0,064257
CC	1	0,027183
MLO	1	0,037074
20181107	2	0,063691
CC	1	0,027602
MLO	1	0,036089
**R**	2	0,066108
20180509	2	0,066108
CC	1	0,028325
MLO	1	0,037783
**5**	11	0,397154
**L**	9	0,331268
20180523	2	0,073102
CC	1	0,036209
MLO	1	0,036893
20180706	3	0,112155
CC	1	0,039455
ML	1	0,035251
MLO	1	0,037449
20180822	2	0,078548
CC	1	0,041069
MLO	1	0,037479
20181114	2	0,067463
CC	1	0,029349
MLO	1	0,038114
**R**	2	0,065886
20180523	2	0,065886
CC	1	0,027253
MLO	1	0,038633
**6**	10	0,279975
**L**	8	0,219664
20180523	2	0,061207
CC	1	0,020323

MLO	1	0,040884
20180703	2	0,065323
CC	1	0,027112
MLO	1	0,038211
20180829	2	0,050345
CC	1	0,022224
MLO	1	0,028121
20181126	2	0,042789
CC	1	0,021277
MLO	1	0,021512
**R**	2	0,060311
20180523	2	0,060311
CC	1	0,020166
MLO	1	0,040145
**7**	10	0,212907
**L**	2	0,051849
20180620	2	0,051849
CC	1	0,022153
MLO	1	0,029696
**R**	8	0,161058
20180620	2	0,04536
CC	1	0,019749
MLO	1	0,025611
20180730	2	0,045055
CC	1	0,019187
MLO	1	0,025868
20181001	2	0,037983
CC	1	0,019851
MLO	1	0,018132
20181210	2	0,03266
CC	1	0,01299
MLO	1	0,01967
**8**	10	0,299345
**L**	4	0,141852
20180711	2	0,069611
CC	1	0,029036
MLO	1	0,040575
20181011	2	0,072241
CC	1	0,030678
MLO	1	0,041563
**R**	6	0,157493
20180711	2	0,062327
CC	1	0,027312
MLO	1	0,035015
20181011	2	0,047819
CC	1	0,020185

MLO	1	0,027634
20181219	2	0,047347
CC	1	0,019627
MLO	1	0,02772
**9**	10	0,326415
**L**	8	0,270001
20180621	2	0,07318
CC	1	0,037031
MLO	1	0,036149
20180727	2	0,053817
CC	1	0,026904
MLO	1	0,026913
20181015	2	0,068165
CC	1	0,040336
MLO	1	0,027829
20181217	2	0,074839
CC	1	0,038253
MLO	1	0,036586
**R**	2	0,056414
20180621	2	0,056414
CC	1	0,028835
MLO	1	0,027579
**10**	10	0,202975
**L**	2	0,04224
20180620	2	0,04224
CC	1	0,0211
MLO	1	0,02114
**R**	8	0,160735
20180620	2	0,037277
CC	1	0,018688
MLO	1	0,018589
20180903	2	0,040733
CC	1	0,019633
MLO	1	0,0211
20181112	2	0,041457
CC	1	0,020357
MLO	1	0,0211
20181219	2	0,041268
CC	1	0,020357
MLO	1	0,020911
**11**	11	0,235326
**L**	2	0,042092
20180704	2	0,042092
CC	1	0,021234
MLO	1	0,020858
**R**	9	0,193234

20180704	2	0,047828
CC	1	0,024277
MLO	1	0,023551
20180726	2	0,041356
CC	1	0,020427
MLO	1	0,020929
20180905	3	0,060778
CC	2	0,040474
MLO	1	0,020304
20181212	2	0,043272
CC	1	0,022736
MLO	1	0,020536
**12**	10	0,31182
**L**	8	0,254909
20180628	2	0,073745
CC	1	0,034063
MLO	1	0,039682
20180717	2	0,05994
CC	1	0,029357
MLO	1	0,030583
20181008	2	0,059019
CC	1	0,029487
MLO	1	0,029532
20181210	2	0,062205
CC	1	0,03113
MLO	1	0,031075
**R**	2	0,056911
20180628	2	0,056911
CC	1	0,024609
MLO	1	0,032302
**13**	13	0,286996
**L**	9	0,195097
20180514	2	0,041437
CC	1	0,019951
MLO	1	0,021486
20180730	3	0,060954
CC	1	0,01985
ML	1	0,021315
MLO	1	0,019789
20181029	2	0,048056
CC	1	0,023764
MLO	1	0,024292
20181203	2	0,04465
CC	1	0,021805
MLO	1	0,022845
**R**	4	0,091899

20180514	2	0,044266
CC	1	0,021717
MLO	1	0,022549
20180730	2	0,047633
CC	1	0,02449
MLO	1	0,023143
**14**	12	0,238786
**L**	2	0,037843
20180711	2	0,037843
MLO	2	0,037843
**R**	10	0,200943
20180711	2	0,038142
CC	1	0,018589
MLO	1	0,019553
20180829	2	0,039085
CC	1	0,019689
MLO	1	0,019396
20181107	2	0,041216
CC	1	0,020399
MLO	1	0,020817
20181221	4	0,0825
CC	2	0,04158
MLO	2	0,04092
**15**	12	0,41058
**L**	2	0,067477
20180801	2	0,067477
CC	1	0,027799
MLO	1	0,039678
**R**	10	0,343103
20180801	3	0,1072
CC	1	0,034996
MLO	2	0,072204
20180903	3	0,097773
CC	2	0,062026
MLO	1	0,035747
20181108	2	0,069123
CC	1	0,042516
MLO	1	0,026607
20190131	2	0,069007
CC	1	0,02934
MLO	1	0,039667
**16**	11	0,265758
**L**	2	0,047452
20180718	2	0,047452
CC	1	0,023831
MLO	1	0,023621

**R**	9	0,218306
20180718	2	0,055273
CC	1	0,033051
MLO	1	0,022222
20180905	3	0,076925
CC	1	0,023044
MLO	2	0,053881
20181107	2	0,045398
CC	1	0,022849
MLO	1	0,022549
20190204	2	0,04071
CC	1	0,020614
MLO	1	0,020096
**17**	10	0,221478
**L**	8	0,173556
20180801	2	0,042393
CC	1	0,017381
MLO	1	0,025012
20180904	2	0,053326
CC	1	0,025675
MLO	1	0,027651
20181025	2	0,036922
CC	1	0,018719
MLO	1	0,018203
20190122	2	0,040915
CC	1	0,020474
MLO	1	0,020441
**R**	2	0,047922
20180801	2	0,047922
CC	1	0,01901
MLO	1	0,028912
**18**	13	0,295622
**L**	10	0,234567
20180907	2	0,037504
CC	1	0,017885
MLO	1	0,019619
20181004	2	0,044309
CC	1	0,01843
MLO	1	0,025879
20181128	3	0,072634
CC	2	0,053392
MLO	1	0,019242
20190129	3	0,08012
CC	2	0,05215
MLO	1	0,02797
**R**	3	0,061055

20180907	3	0,061055
CC	2	0,040393
MLO	1	0,020662
**19**	11	0,34527
**L**	2	0,063888
20180801	2	0,063888
CC	1	0,031982
MLO	1	0,031906
**R**	9	0,281382
20180801	2	0,065946
CC	1	0,027702
MLO	1	0,038244
20181003	2	0,060176
CC	1	0,032201
MLO	1	0,027975
20181120	2	0,060948
CC	1	0,031357
MLO	1	0,029591
20181211	3	0,094312
CC	2	0,063533
MLO	1	0,030779
**21**	8	0,16498
**L**	2	0,042621
20180912	2	0,042621
CC	1	0,0211
MLO	1	0,021521
**R**	6	0,122359
20180912	2	0,038219
CC	1	0,01887
MLO	1	0,019349
20181113	2	0,042263
CC	1	0,021107
MLO	1	0,021156
20190213	2	0,041877
CC	1	0,020625
MLO	1	0,021252
**22**	11	0,298153
**L**	2	0,051347
20180912	2	0,051347
CC	1	0,02122
MLO	1	0,030127
**R**	9	0,246806
20180912	2	0,049876
CC	1	0,017321
MLO	1	0,032555
20181030	2	0,053545

CC	1	0,027879
MLO	1	0,025666
20190107	3	0,079033
CC	2	0,05305
MLO	1	0,025983
20190304	2	0,064352
CC	1	0,026942
MLO	1	0,03741
**23**	10	0,311547
**L**	8	0,263415
20180926	2	0,075522
CC	1	0,037698
MLO	1	0,037824
20181128	2	0,063745
CC	1	0,02613
MLO	1	0,037615
20190124	2	0,062128
CC	1	0,026085
MLO	1	0,036043
20190411	2	0,06202
CC	1	0,02515
MLO	1	0,03687
**R**	2	0,048132
20180926	2	0,048132
CC	1	0,020476
MLO	1	0,027656
**24**	10	0,213149
**L**	8	0,173109
20180926	2	0,053603
CC	1	0,017921
MLO	1	0,035682
20181115	2	0,037794
CC	1	0,019044
MLO	1	0,01875
20190128	2	0,041782
CC	1	0,020471
MLO	1	0,021311
20190422	2	0,03993
CC	1	0,0198
MLO	1	0,02013
**R**	2	0,04004
20180926	2	0,04004
CC	1	0,020659
MLO	1	0,019381
**25**	10	0,192925
**L**	8	0,154006

20181010	2	0,03577
CC	1	0,017885
MLO	1	0,017885
20181123	2	0,038912
CC	1	0,019279
MLO	1	0,019633
20190204	2	0,040714
CC	1	0,020357
MLO	1	0,020357
20190422	2	0,03861
CC	1	0,01895
MLO	1	0,01966
**R**	2	0,038919
20181010	2	0,038919
CC	1	0,018934
MLO	1	0,019985
**26**	10	0,28101
**L**	8	0,223411
20181107	2	0,063952
CC	1	0,027901
MLO	1	0,036051
20190111	2	0,068199
CC	1	0,039071
MLO	1	0,029128
20190329	2	0,04327
CC	1	0,02086
MLO	1	0,02241
20190516	2	0,04799
CC	1	0,02037
MLO	1	0,02762
**R**	2	0,057599
20181107	2	0,057599
CC	1	0,031687
MLO	1	0,025912
**27**	9	0,266893
**L**	9	0,266893
20181213	2	0,062585
CC	1	0,029509
MLO	1	0,033076
20190104	3	0,105168
CC	1	0,021979
MLO	2	0,083189
20190218	2	0,04659
CC	1	0,021715
MLO	1	0,024875
20190524	2	0,05255

CC	1	0,02125
MLO	1	0,0313
**28**	10	0,243355
**L**	8	0,201847
20181024	2	0,044788
CC	1	0,025706
MLO	1	0,019082
20181122	2	0,045048
CC	1	0,017715
MLO	1	0,027333
20190207	2	0,054717
CC	1	0,026905
MLO	1	0,027812
20190315	2	0,057294
CC	1	0,028965
MLO	1	0,028329
**R**	2	0,041508
20181024	2	0,041508
CC	1	0,020494
MLO	1	0,021014
**29**	13	0,314116
**L**	11	0,265771
20181024	3	0,077819
CC	1	0,019602
MLO	2	0,058217
20190108	3	0,077528
CC	1	0,027991
ML	1	0,029075
MLO	1	0,020462
20190313	3	0,070204
CC	1	0,020066
ML	1	0,020708
MLO	1	0,02943
20190531	2	0,04022
CC	1	0,01969
MLO	1	0,02053
**R**	2	0,048345
20181024	2	0,048345
CC	1	0,020013
MLO	1	0,028332
**30**	10	0,250251
**L**	2	0,041686
20181127	2	0,041686
CC	1	0,021836
MLO	1	0,01985
**R**	8	0,208565

20181127	2	0,043138
CC	1	0,018157
MLO	1	0,024981
20190117	2	0,052427
CC	1	0,02703
MLO	1	0,025397
20190314	2	0,05468
CC	1	0,019781
MLO	1	0,034899
20190513	2	0,05832
CC	1	0,01996
MLO	1	0,03836
**31**	11	0,220447
**L**	9	0,17787
20181205	3	0,058127
CC	1	0,019279
MLO	2	0,038848
20190103	2	0,040697
CC	1	0,020458
MLO	1	0,020239
20190314	2	0,039636
CC	1	0,019075
MLO	1	0,020561
20190528	2	0,03941
CC	1	0,01937
MLO	1	0,02004
**R**	2	0,042577
20181205	2	0,042577
CC	1	0,021096
MLO	1	0,021481
**32**	10	0,201718
**L**	2	0,039994
20181107	2	0,039994
CC	1	0,020064
MLO	1	0,01993
**R**	8	0,161724
20181107	2	0,038715
CC	1	0,019317
MLO	1	0,019398
20190206	2	0,039439
CC	1	0,019942
MLO	1	0,019497
20190409	2	0,03819
CC	1	0,02026
MLO	1	0,01793
20190701	2	0,04538

CC	1	0,01917
MLO	1	0,02621
**33**	11	0,256641
**L**	2	0,037804
20181214	2	0,037804
CC	1	0,019101
MLO	1	0,018703
**R**	9	0,218837
20181214	2	0,046482
CC	1	0,019977
MLO	1	0,026505
20190130	2	0,046455
CC	1	0,020274
MLO	1	0,026181
20190402	3	0,07288
CC	2	0,03773
MLO	1	0,03515
20190627	2	0,05302
CC	1	0,02609
MLO	1	0,02693
**34**	10	0,223396
**L**	8	0,178946
20181212	2	0,053856
CC	1	0,02595
MLO	1	0,027906
20190117	2	0,046575
CC	1	0,019356
MLO	1	0,027219
20190306	2	0,039935
CC	1	0,019773
MLO	1	0,020162
20190515	2	0,03858
CC	1	0,01885
MLO	1	0,01973
**R**	2	0,04445
20181212	2	0,04445
CC	1	0,019095
MLO	1	0,025355
**35**	10	0,254791
**L**	8	0,197768
20181024	2	0,063969
CC	1	0,036899
MLO	1	0,02707
20190110	2	0,038929
CC	1	0,019509
MLO	1	0,01942

20190328	2	0,05189
CC	1	0,02083
MLO	1	0,03106
20190520	2	0,04298
CC	1	0,02254
MLO	1	0,02044
**R**	2	0,057023
20181024	2	0,057023
CC	1	0,028448
MLO	1	0,028575
**36**	8	0,159329
**L**	6	0,116652
20181121	2	0,040406
CC	1	0,020029
MLO	1	0,020377
20190201	2	0,041546
CC	1	0,020991
MLO	1	0,020555
20190614	2	0,0347
CC	1	0,01315
MLO	1	0,02155
**R**	2	0,042677
20181121	2	0,042677
CC	1	0,021283
MLO	1	0,021394
**37**	9	0,289056
**L**	9	0,289056
20181228	2	0,065462
CC	1	0,033084
MLO	1	0,032378
20190211	2	0,071874
CC	1	0,037042
MLO	1	0,034832
20190326	3	0,09549
CC	1	0,02866
ML	1	0,02716
MLO	1	0,03967
20190527	2	0,05623
CC	1	0,0281
MLO	1	0,02813
**38**	8	0,237033
**L**	8	0,237033
20190109	2	0,064538
CC	1	0,027885
MLO	1	0,036653
20190208	2	0,064715

CC	1	0,02713
MLO	1	0,037585
20190322	2	0,06658
CC	1	0,02744
MLO	1	0,03914
20190710	2	0,0412
CC	1	0,02031
MLO	1	0,02089
**39**	16	0,329733
**L**	2	0,040113
20190102	2	0,040113
CC	1	0,019644
MLO	1	0,020469
**R**	14	0,28962
20190102	3	0,067538
CC	2	0,043887
MLO	1	0,023651
20190123	5	0,104142
CC	2	0,037739
ML	1	0,02545
MLO	2	0,040953
20190411	4	0,07281
CC	1	0,01836
ML	1	0,01892
MLO	2	0,03553
20190719	2	0,04513
CC	1	0,0191
MLO	1	0,02603
**D**	11	0,239408
**L**	10	0,220129
20180704	3	0,058958
CC	2	0,034481
MLO	1	0,024477
20180827	7	0,161171
CC	4	0,097213
MLO	3	0,063958
**R**	1	0,019279
20180704	1	0,019279
MLO	1	0,019279
**Total**	405	10,403037

MLO – mediolateral oblique view; CC – craniocaudal view; R – right; L - left; MGD_LE count - mean average glandular dose count of low energy images; MGDtotal sum - mean average glandular dose count of CEM
